# Is MTHFD1 polymorphism rs 2236225 (c.1958G>A) associated with the susceptibility of NSCL/P? A systematic review and meta-analysis

**DOI:** 10.12688/f1000research.6425.2

**Published:** 2016-01-06

**Authors:** Huaxiang Zhao, Jieni Zhang, Mengqi Zhang, Feng Deng, Leilei Zheng, Hui Zheng, Feng Chen, Jiuxiang Lin

**Affiliations:** 1 Department of Orthodontics, Peking University School and Hospital of Stomatology, Peking, 100081, China; 2 Bybo Dental Group, Beijing, 100062, China; 3 Department of Orthodontics, Affiliated Hospital of Stomatology, Chongqing Medical University, Chongqing, 401147, China; 4 Laboratory Center, Peking University School and Hospital of Stomatology, Peking, 100081, China

**Keywords:** MTHFD1, Polymorphisms, NSCL/P susceptibility, Meta-analysis

## Abstract

**Aims:** To investigate the association between the methylenetetrahydrofolate dehydrogenase 1 (MTHFD1) polymorphism rs 2236225 (c.1958G>A) and susceptibility to non-syndromic cleft of the lip and/or palate (NSCL/P).

**Methods:** An extensive literature review has been conducted using PubMed, Web of Science, Cochrane Library, Google Scholar, the China National Knowledge Infrastructure (CNKI), and Wanfang Database for eligible researches. The terms for searching were “cleft lip OR cleft palate OR CLP OR CL/P OR oral facial cleft OR OFC” AND “methylenetetrahydrofolate dehydrogenase (NADP+ dependent) 1 OR methenyltetrahydrofolate cyclohydrolase formyltetrahydrofolate synthetase OR MTHFD1 OR MTHFD”. Two independent researchers screened, evaluated and extracted the data of included studies. The pooled odds ratios (OR) with 95% confidence intervals (95% CI) were calculated by random effects model under five gene models. Subgroup, sensitivity analysis and publication bias were also assessed.

**Results:** Ten case-control studies have been included in the systematic review and eight studies have been considered for the meta-analysis. Overall, the MTHFD1 polymorphism rs2236225 and the risk of NSCL/P showed pooled OR (95% CI) of 1.02 (0.86-1.21) under allelic model. A higher degree of heterogeneity was observed in Asian countries (I ^2^ = 75.6%) compared to non-Asian countries (I ^2^ = 48.9%). Similar consequence appeared in the subgroup of children (I ^2^ = 78.6%) compared with that of mothers (I ^2^ = 0.0%). There was no significant difference in the publication bias by the Begg’s funnel plot (P = 0.711) and Egger’s regression test (P = 0.746).

**Conclusion:** Our assessment suggested there was no significant association between the MTHFD1 polymorphism rs 2236225 (c.1958G>A) and the susceptibility to NSCL/P. Further investigations using a large sample size and a more advanced technique should be adopted to reach a more precise conclusion in the future.

## Introduction

Cleft of the lip and/or palate (CL/P) is one of the most common facial malformations ^1– 3^ and a societal burden, affecting the patient ability to eat and speak and influencing social integration ^4^. Non-syndromic CL/P, accounting for about 70% of CL/P, is considered closely related to genetic and environmental factors ^5^. Recent studies suggested that using folic acid could reduce the rates of oral clefts ^6, 7^ and single nucleotide polymorphisms of some genes such as MTHFR ^8, 9^, MTR ^40^ and MTRR involved in the metabolism of folic acid have been associated to high risk of NSCL/P ^8, 9^. Methylenetetrahydrofolate dehydrogenase 1 ( *MTHFD1*), a key gene associated with three sequential enzymatic reactions in the metabolism of folic acid, might play a potential role in the risk of NSCL/P, especially the polymorphism rs2236225 (c.1958G>A) ^10^. Indeed, different observations that linked the polymorphism rs2236225 to the risk of NSCL/P have been reported ^11, 12^. The suggestion of a link between rs2236225 polymorphism and susceptibility to NSCL/P might be result of the limitations in sample size, different ethnic populations and other environmental factors. Therefore, we conducted a systematic review and meta-analysis of eligible case-control studies to reveal a more precise connection between the *MTHFD1* polymorphism rs2236225 and the risk of NSCL/P.

## Materials and methods

### Identification of studies

A systematic search based on the principle of evidence-based medicine ^13^ was performed in PubMed, Web of Science, Cochrane Library, Google Scholar, China National Knowledge Infrastructure (CNKI) and WanFang Database. The final update was made on April 5th, 2015. In line with our knowledge background, the Medical Subject Headings (MESH) terms in PubMed and the known aliases of the genes of interests in the National Center of Biotechnology Information (NCBI), the following terms were used for searching: “cleft lip OR cleft palate OR CLP OR CL/P OR oral facial cleft OR OFC” AND “methylenetetrahydrofolate dehydrogenase (NADP+ dependent) 1 OR methenyltetrahydrofolate cyclohydrolase formyltetrahydrofolate synthetase OR *MTHFD1* OR MTHFD”, which were slightly adjusted to optimize search results ( Table S1; PubMed). We didn’t limit the search depending on publication types, data and language. Of course, the review of the published literature was examined carefully and manual search was conducted to avoid missing potential data. Two of the authors (Huaxiang Zhao and Mengqi Zhang) were in charge of the search independently and a third author (Jieni Zhang) conducted a random inspection.

### Inclusion and exclusion criteria

Researches included in our systematic review and meta-analysis meet the following criteria: (1) evaluating the association between the NSCL/P and *MTHFD1* polymorphism rs2236225, (2) focusing on humans, (3) case-control studies. Exclusion criteria were: (1) no association between NSCL/P and MTFHD1, (2) not focusing on humans but animal models or *in vitro* studies, (3) duplication of previous researches, (4) not original literature such as reviews, meta-analyses, comments and editorials.

### Data collection

Data from eligible studies were extracted by two independent researchers (Huaxiang Zhao and Mengqi Zhang) in accordance with the inclusion and exclusion criteria. In case of any discrepancies, the third chief author (Feng Chen) would make a further investigation or bring it into a group-discussion. A special table was used for collecting information from the selected articles and the following entries were recorded: authors (year), country, location of geography, subjects, methods for genotyping, sample size of cases/controls, descriptions of samples rolled in the study, P for HWE (Hardy-Weinberg equilibrium) of control group, whether included in the meta-analysis or not.

### Methodological quality assessment

A methodological quality assessment adapted from previous studies ^14– 16^ was carried on included studies ( Table S2). Cases, source of controls, sample sizes and Hardy-Weinberg equilibrium (HWE) were considered as important aspects in this systematic review. It is not a widely-used standard so its efficiency is not certain. The result does not include the judgments but the standard can be a reference for the certainty of conclusion.

### Statistical analysis

The PRISMA checklist ( Supplementary material S3) was used as a protocol in our meta-analysis ^17^. Odd ratios (ORs) and 95% confidence intervals (CIs) were calculated to estimate the association between the susceptibility to NSCL/P and *MTHFD1*. Five genetic models were used in the process of pooling the OR and 95% CIs: allelic comparison (A versus G), heterozygote model (AG versus GG), homozygote model (AA versus GG), dominant model (AA + AG versus GG), recessive model (AA versus AG + GG). The significance of the pooled effects was determined by Z-test with P value less than 0.05. The Q-statistic and the I ^2^ test were used to evaluated; P < 0.05 in Q statistic or I ^2^ > 50% ^18, 19^, would indicate a significant heterogeneity. When P > 0.05 in Q statistic or I ^2^ < 50%, the fixed pooling model (Mantel-Haenszel) was conducted; if not, the random pooling model (M-H heterogeneity) was used. We also carried subgroup analyses in which different subjects (mothers or children), location of geography (non-Asian countries or Asian countries) were considered potential source of heterogeneity. A sensitivity analysis was conducted by omitting each study in turn to evaluate the single study’s influence on the overall estimation. We used Begg’s funnel plot and Egger’s linear regression test to find out the publication bias of the included studies ^20– 22^. The studies with disequilibrium of HWE among control group were added into a supplementary meta-analysis as described previously ^23^. Meanwhile, as for the studies included but not carried into the meta-analysis, to achieve a qualitative analysis we adopted a method described by others ^24^. Results were considered significant when P < 0.05. Stata 12.0 (Stata Corp, College Station, TX, USA) was used for the analysis.

## Results

### Data retrieval

**Figure 1.**
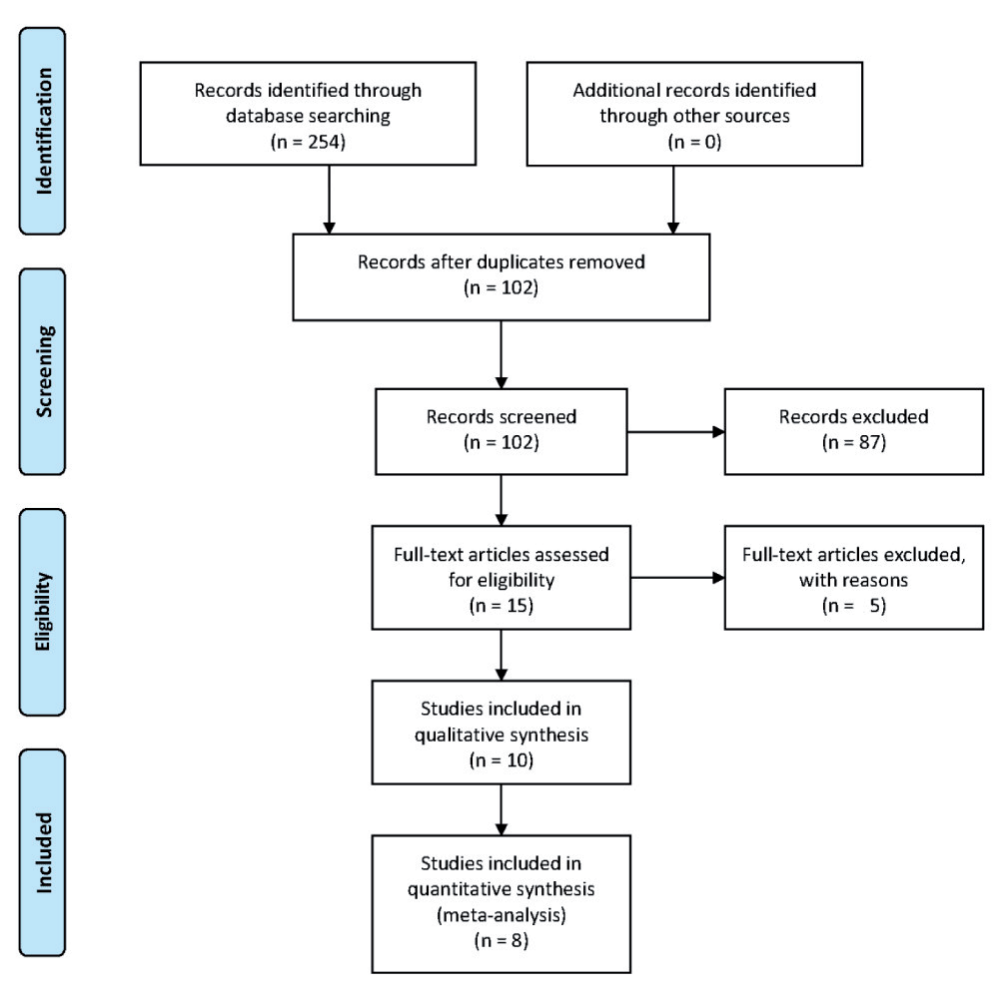
Flow chart showing study selection in the systematic and meta-analysis.

A total of 251 articles resulted from the search described above (PubMed: 86, Web of Science: 8, Google Scholar: 135, Cochrane Library: 0, CNKI: 18, Wanfang: 4). After being imported into EndNote X6 software (Thomson Corporation, Stamford), a screening process was conducted among 102 articles– that is, duplicates were removed using the ‘Discard Duplicates’ function as well as by handwork. Following paper selection by two independent researchers, 15 studies were then thoroughly reviewed. Of these, five studies were excluded, among which two had no control groups ^25, 26^, one no relation to MTFHD1 ^27^, and the other two presented data previously published ^28, 29^. Finally, 10 studies that met the criteria were included in the systematic review ( Table 1) ^10– 12, 30– 36^ and mathematic data from eight studies were used for reference to carry out the meta-analysis ^10– 12, 31– 33, 35, 36^. The selection process is shown in Figure 1.

**Table 1.** Characteristics of studies included in the systematic review and meta-analysis.

No.	Authors (year)	Country	Geographical location	Subjects	Methods for genotyping	Sample size of case/control group (just for the patients)		Descriptions of samples from study participants	P for HWE* of control group	Whether included in meta- analysis or not
						case	control			
1	Mostowska *et al.* (2006)	Poland	Europe	Mothers	PCR-RFLP ^γ^	122	82	The case samples came from healthy mothers of NSCL/P children, while the control group includes samples from healthy mothers of children without NSCL/P. There was no difference between the two groups in their age, habit of smoking.	NM ^ψ^	Yes
2	Boyles *et al.* (2008)	Norway	Europe	Mothers and children	MALDI-TOF MS ^ξ^	573	763	377 cases were CL/P and 196 cases CPO. Most mothers in the case group use supplemental folate during the pregnancy.	NM ^ψ^	No
3	Mills *et al.* (2008)	Ireland	Europe	Mothers, fathers and children	PCR-RFLP ^γ^	1030	1000	536 were CLP consisted of 494 cases with isolated defects 23 with one additional defect, 18 with multiple defects, and one with Pierre Robin. 426 cases with CPO consisted of 321 isolated defects, 15 with one additional defect, 21 with multiple defects, and 69 with Pierre Robin Sequence.	0.03	Yes
4	Bufalino *et al.* (2010)	Brazil	South America	Mothers	PCR-RFLP ^γ^	106	184	Mothers who smoke, drink and use anti- hypertensives and drugs that could potentially impair the function of folic acids were not included in this study.	0.66	Yes
5	Mostowska *et al.* (2010)	Poland	Europe	Children	PCR-RFLP ^γ^	174	176	The patients with clefts palate only (CPO) were excluded because the researchers thought the pathogenesis of NSCL/P and the CPO was different.	0.11	Yes
6	Li *et al.* (2013)	China	Asian	Children	PCR-RFLP ^γ^	187	157	The patients in the case group consisted of 126 boys and 61 girls.	0.89	Yes
7	Yuan (2013)	China	Asian	Mothers, fathers and children	PCR-RFLP ^γ^	150	150	68 CLO and 82 CLP were enrolled in the case group.	0.92	Yes
8	Zhao *et al.* (2013)	China	Asian	Children	PCR-RFLP ^γ^	294	126	There were 191 CLP and 103 CPO in the patients group.	0.08	Yes
9	de Aquino *et al.* (2013)	Brazil	South America	Mothers, fathers and children	Real-Time PCR	181	478	Patients with clefts palate only (CPO) were excluded. 65 clefts lip only (CLO) and 116 clefts lip and palate (CLP) were included in this study, consisting of 101 males and 80 females.	NM ^ψ^	No
10	Murthy *et al.* (2014)	India	Asian	Children	PCR-RFLP ^γ^	142	141	There were 123 CLP and 19 CPO in the case group.	0.94	Yes

HWE*: Hardy-Weinberg equilibrium.

NM ^ψ^: Not mentioned in the study.

PCR-RFLP ^γ^: PCR-restriction fragment length polymorphism.

MALDI-TOF MS ^ξ^: matrix-assisted laser desorption/ionization time-of-flight mass spectrometry.

### Study characteristics

Eventually, all 10 studies containing 6216 samples (2959 cases and 3257 controls) were analyzed in our review. The characteristics of every study can be seen in Table 1. To summarize briefly, there were four studies from European groups, four from Asian groups and two from South American groups, among which two studies focused on the genotype of patients’ mothers only, four on children’ s genotype only and four on both of them. PCR-restriction fragment length polymorphism (PCR-RFLP) was the major method of genotyping, while other techniques had been used as well.

### Association between *MTHFD1* polymorphism rs2236225 (c.1958G>A) and NSCL/P susceptibility

**Figure 2.**
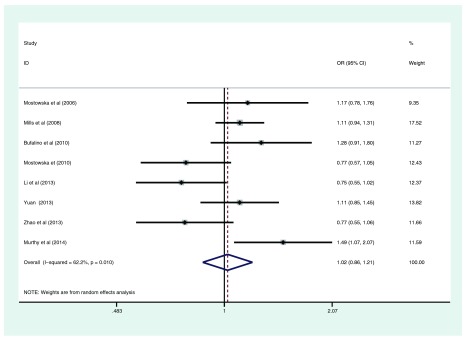
Forest plot of allelic comparison of *MTHFD1* polymorphism rs2236225 (c.1958G>A) for overall comparison (A versus G).

**Figure 3.**
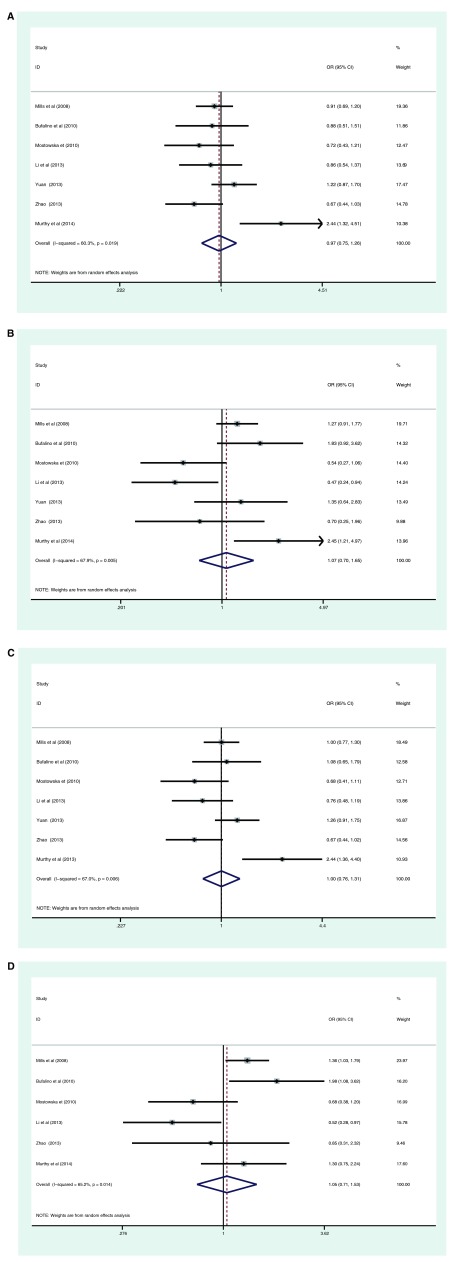
Forest plot of heterozygote, homozygote, dominant and recessive model comparison of *MTHFD1* polymorphisms rs2236225 (c.1958G>A) for overall comparison. ( **A**) Heterozygote model, AG versus GG. ( **B**) Homozygote model, AA versus GG. ( **C**) Dominant model, AA + AG versus GG. ( **D**) Recessive model, AA versus AG + GG.

The association between *MTHFD1* polymorphism rs2236225 (c.1958G>A) and NSCL/P susceptibility was analyzed through a meta-analysis and qualitative analysis. In the meta-analysis, since significant heterogeneity had been identified by Q-test and I ^2^ statistic in every genetic model, the random effect models were used. Overall, a significant association was not found in any genetic model (A versus G: OR = 1.02, 95% CI 0.86–1.21, P _H_ = 0.010, Figure 2; AG versus GG: OR = 0.97, 95% CI 0.75–1.26, P _H_ = 0.019, Figure 3A; AA versus GG: OR = 1.07, 95% CI 0.70–1.65, P _H_ = 0.005, Figure 3B; AA + AG versus GG: OR = 1.00, 95% CI 0.76–1.31, P _H_ = 0.006, Figure 3C; AA versus AG + GG: OR = 1.05, 95% CI 0.71–1.53, P _H_ = 0.014, Figure 3D). On the other hand, no association was found in the genotypes of children, mothers or fathers in the qualitative analysis ^30, 34^.

**Figure 4.**
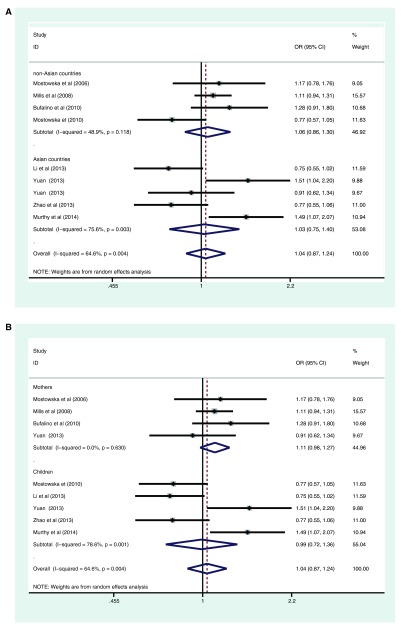
Subgroup analysis by locations of geography ( **A**) and subjects ( **B**) under allelic comparison of *MTHFD1* polymorphism rs2236225 (c.1958G>A).

Next we conducted the subgroup analysis using allelic A versus G model according to the location of geography and subjects (mothers or children). It turned out that there was no significant difference between Asian (OR = 1.03, 95% CI 0.75–1.40, P _H_ = 0.003) or non-Asian population (OR = 1.06, 95% CI 0.86–1.30, P _H_ = 0.118). However, a higher degree of heterogeneity was observed in the Asian countries compared to non-Asian countries ( Figure 4A). A similar result was observed in the subgroup analysis between mothers and children. The heterogeneity was much higher in the children group (OR = 0.99, 95% CI 0.72–1.36, P _H_ = 0.001) than in the mothers’ group (OR = 1.11, 95% CI 0.98–1.27, P _H_ = 0.630), while no significant difference was observed in both groups ( Figure 4B).

### Sensitivity analysis and publication bias

**Figure 5.**
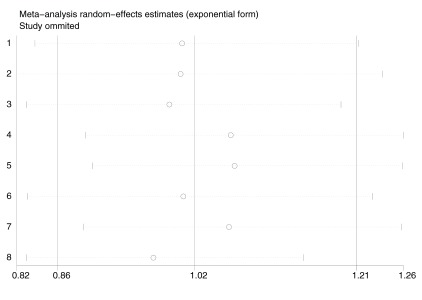
Sensitivity analysis of the association between *MTHFD1* polymorphism rs2236225 (c.1958G>A) and susceptibility to NSCL/P under allelic model (A versus G).

To access the influence of each individual study on the pooled ORs, a sensitivity analysis was performed by omitting each study at a time. The results of sensitivity suggests that no individual study affects the pooled ORs of the associations between *MTHFD1* polymorphism rs2236225 (c.1958G>A) and NSCL/P risk under allelic model ( Figure 5).

**Figure 6.**
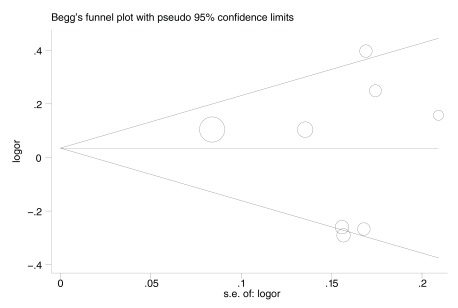
Begg’s funnel plot of the association between *MTHFD1* polymorphism rs2236225 (c.1958G>A) and the susceptibility to NSCL/P under allelic model (A versus G).

We used the Begg’s funnel plot and Egger’s regression test (both used the allelic A versus G model) to estimate the publication bias. Our results indicate that there is no significant publication bias both in the symmetry of Begg’s funnel plot (P = 0.711, Figure 6) and Egger’s regression test (P = 0.746). But due to the sample size (the total number of included article is less than 10), statistical analyses may not describe the publication bias precisely. The test reliability is in doubt.

## Discussion

CL/P is one of the most common facial malformations, affecting approximately 1.7/1000 people around the world with ethnic and geographic variation ^1^. Approximately 70% of CL/P cases are considered to be non-syndromic ^37, 38^, and their susceptibility has been linked to the expression of various candidate genes through twin studies, familial clustering studies and genome-wide studies ^39^. Recent studies suggest that using folic acid could reduce the rates of oral clefts ^6, 7^ and genes involved in the metabolish of folic acid have been identified *MTHFD1*, a crucial gene associated with three sequential enzymatic reactions among 5,10-methylenetetrahydrofolate, 5,10-methenyltetrahydrofolate, 10-formyltetrahydrofolate, tetrahydrofolate, might play a potential role in NSCL/P ^10^. However, controversial results about the *MTHFD1* polymorphism rs2236225 (c.1958G>A) have been reported in different articles ^10, 12^.

In this systematic review, 10 independent case-control studies were included (eight studies for meta-analysis and two studies qualitatively analyzed) containing 6216 samples (2959 cases and 3257 controls). In all of the included 8 studies for meta-analysis, 7 did not show significant difference between the case and control groups. 1 study reported that the case group showed closer relationship with *MTHFD1* polymorphisms rs 2236225 (c.1958G>A). After the over-all analysis we concluded the comprehensive effect. All the eligible studies of meta-analysis and qualitative analysis showed no significant association of *MTHFD1* rs2236225 to the risk of NSCL/P, whether in the whole analysis of five model (A versus G, AG versus GG, AA versus GG, AA + AG versus GG, AA versus AG + GG) or in the subgroup of subjects (mothers or children) and the location of geography (non-Asian countries or Asian countries). Meanwhile, high heterogeneity was observed, which might be the reason for the genetic drift and natural selection among different ethnic groups ^42^. Also, small sample size of different studies might be a possible reason for the disparate results. Our findings suggest that the *MTHFD1* polymorphism rs2236225 (c.1958G>A) might not be an appropriate biomarker in predicting the susceptibility of an individual to NSCL/P.

Some limitations of this systematic review and meta-analysis should be noted. Firstly, the choice of retrospective studies has its own limitations, as we may encounter selection bias and influence the results of our analysis ^43^. However, a bigger size of cohort study cannot be conducted easily because of the relatively low morbidity ^44^. Secondly, only 10 studies were included in our review, a small sample size that might not provide sufficient evidence to estimate the connections between the *MTHFD1* polymorphisms and the risk of NSCL/P. Thirdly, the publication bias cannot be effectively analyzed because of the limited amount of included study.

NSCL/P is also associated with gene-gene and gene-environment interactions ^45^. Although no correlation was observed between MTFHD1 polymorphism rs2236225 (c.1958G>A) and the risk of NSCL/P, in view of MTFHD1 gene’s key role in folic acid metabolism, we cannot draw a definite conclusion that there is no association between MTFHD1 and NSCL/P’s susceptibility. The use of larger sample size studies, different techniques and considering gene-gene or gene-environment interactions should be explored in future investigations. What is more, the gene samples from mother were too scarce to be representative and to explain our results. We do recommend more samples from parents in the future studies, which is significant for the early stage diagnose, as the current technology can only diagnose CLP in the midterm even later in the pregnancy.

## Figures and Tables

**Table S1.** Typical search terms used in Pubmed.

#1	cleft lip
#2	cleft palate
#3	cleft lip and palate
#4	cleft lip and/or palate
#5	CLP
#6	CL/P
#7	oral facial cleft
#8	OFC
#9	methylenetetrahydrofolate dehydrogenase (NADP+ dependent) 1
#10	methenyltetrahydrofolate cyclohydrolase formyltetrahydrofolate synthetase
#11	*MTHFD1*
#12	*MTHFD*
#13	*MTHFC*
#14	#1 OR #2 OR #3 OR #4 OR #5 OR #6 OR #7 OR #8
#15	#9 OR #10 OR #11 OR #12 OR #13
#16	#14 AND #15

**Table S2.** Scale for methodological quality assessment.

Items	Score
1. Representative cases	
NSCL/P diagnosed by acknowledged criteria.	2
Mentioned the diagnosed criteria but not described specifically.	1
Not Mentioned.	0
2. Source of controls	
Population or community-based	2
Hospital-based	1
Not described	0
3. Sample size	
>300	2
150–300	1
<150	0
4. Hardy-Weinberg equilibrium (HWE)	
Hardy-Weinberg equilibrium in control group	1
Hardy-Weinberg disequilibrium in control group	0

**Table S3.** PRISMA checklist used for protocol (available at http://www.prisma-statement.org/statement.htm).

Section/topic	#	Checklist item	Reported on page #
Title			
Title	1	Identify the report as a systematic review, meta-analysis, or both.	1
**Abstract**			
Structured summary	2	Provide a structured summary including, as applicable: background; objectives; data sources; study eligibility criteria, participants, and interventions; study appraisal and synthesis methods; results; limitations; conclusions and implications of key findings; systematic review registration number.	2~3
**Introduction**			
Rationale	3	Describe the rationale for the review in the context of what is already known.	4
Objectives	4	Provide an explicit statement of questions being addressed with reference to participants, interventions, comparisons, outcomes, and study design (PICOS).	5
**Methods**			
Protocol and registration	5	Indicate if a review protocol exists, if and where it can be accessed (e.g., Web address), and, if available, provide registration information including registration number.	none
Eligibility criteria	6	Specify study characteristics (e.g., PICOS, length of follow-up) and report characteristics (e.g., years considered, language, publication status) used as criteria for eligibility, giving rationale.	5~6
Information sources	7	Describe all information sources (e.g., databases with dates of coverage, contact with study authors to identify additional studies) in the search and date last searched.	5
Search	8	Present full electronic search strategy for at least one database, including any limits used, such that it could be repeated.	Table S1
Study selection	9	State the process for selecting studies (i.e., screening, eligibility, included in systematic review, and, if applicable, included in the meta-analysis).	5~6
Data collection process	10	Describe method of data extraction from reports (e.g., piloted forms, independently, in duplicate) and any processes for obtaining and confirming data from investigators.	5~6
Data items	11	List and define all variables for which data were sought (e.g., PICOS, funding sources) and any assumptions and simplifications made.	5~6
Risk of bias in individual studies	12	Describe methods used for assessing risk of bias of individual studies (including specification of whether this was done at the study or outcome level), and how this information is to be used in any data synthesis.	6 and Table S2
Summary measures	13	State the principal summary measures (e.g., risk ratio, difference in means).	6~7
Synthesis of results	14	Describe the methods of handling data and combining results of studies, if done, including measures of consistency (e.g., I ^2^) for each meta-analysis.	6~7
Risk of bias across studies	15	Specify any assessment of risk of bias that may affect the cumulative evidence (e.g., publication bias, selective reporting within studies).	6~7
Additional analyses	16	Describe methods of additional analyses (e.g., sensitivity or subgroup analyses, meta-regression), if done, indicating which were pre-specified.	7
**Results**			
Study selection	17	Give numbers of studies screened, assessed for eligibility, and included in the review, with reasons for exclusions at each stage, ideally with a flow diagram.	8 and Figure 1
Study characteristics	18	For each study, present characteristics for which data were extracted (e.g., study size, PICOS, follow-up period) and provide the citations.	Table 1
Risk of bias within studies	19	Present data on risk of bias of each study and, if available, any outcome level assessment (see item 12).	9~10, Table S2
Results of individual studies	20	For all outcomes considered (benefits or harms), present, for each study: (a) simple summary data for each intervention group (b) effect estimates and confidence intervals, ideally with a forest plot.	Figure 2
Synthesis of results	21	Present results of each meta-analysis done, including confidence intervals and measures of consistency.	Figure 2~ Figure 4
Risk of bias across studies	22	Present results of any assessment of risk of bias across studies (see Item 15).	Table S2, Figure 5~ Figure 6
Additional analysis	23	Give results of additional analyses, if done (e.g., sensitivity or subgroup analyses, meta-regression [see Item 16]).	Figure 5~ Figure 6
**Discussion**			
Summary of evidence	24	Summarize the main findings including the strength of evidence for each main outcome; consider their relevance to key groups (e.g., healthcare providers, users, and policy makers).	11~12
Limitations	25	Discuss limitations at study and outcome level (e.g., risk of bias), and at review-level (e.g., incomplete retrieval of identified research, reporting bias).	12
Conclusions	26	Provide a general interpretation of the results in the context of other evidence, and implications for future research.	13
**Funding**			
Funding	27	Describe sources of funding for the systematic review and other support (e.g., supply of data); role of funders for the systematic review.	15
