# Supplementary material for: Is MTHFD1 polymorphism rs 2236225 (c.1958G>A) associated with the susceptibility of NSCL/P? A systematic review and meta-analysis
Source: F1000Res. 2016 Jan 6;4:142. Originally published 2015 Jun 4. [Version 2] doi: 10.12688/f1000research.6425.2 (PMC4722688; doi:10.12688/f1000research.6425.2)
Supplement: Peer review history

# Peer review: Referee response for version 2

**DOI:** 10.5256/f1000research.7371.r11833

**Published:** 2016-01-14

**Authors:** Jose Suazo

**Affiliations:** 1 Institute for Research in Dental Sciences, Faculty of Dentistry, University of Chile, Santiago, Chile

The current version of this report has properly included all of my suggestions and comments. I think that now this version was really improved the message for the readers.

I have read this submission. I believe that I have an appropriate level of expertise to confirm that it is of an acceptable scientific standard.

# Peer review: Referee response for version 1

**DOI:** 10.5256/f1000research.6892.r9328

**Published:** 2015-07-06

**Authors:** Jose Suazo

**Affiliations:** 1 Institute for Research in Dental Sciences, Faculty of Dentistry, University of Chile, Santiago, Chile

In general terms, this article contributes to unraveling the complex genetic architecture of NSCL/P. This type of systematic reviews are always relevant due to the fact that they pooled several singles trials, and therefore, the sample sizes have an important increase. However, this article needs some modifications that avoid to be published in its current version:

1. All of the gene acronyms may be written italics.
2. The title says "Is MTHFD1 polymorphisms..." but it refers to only one SNP.
3. The Materials and Method section includes the quality analysis of each paper included in this meta-analysis, but Results section did not mentioned anything about this analysis.
4. The publication bias analysis when < 10 articles is not statistically resolved (see Egers's paper), so for this meta-analysis it is necessary at least a comment about this issue.
5. The first two paragraph of the Discussion section are almost the same as the Introduction.
6. I think that the Discussion could include a comment about maternal genotype effects in order to explain the negative results of this analysis.

I have read this submission. I believe that I have an appropriate level of expertise to confirm that it is of an acceptable scientific standard, however I have significant reservations, as outlined above.

# Response

**Published:** 2015-11-26

**Authors:** Feng Chen

**Affiliations:** School of Stomatology, Peking University, China

Thank you so much for your precious advice. We have studied your comments carefully and revised the manuscript. The point to point responds are listed as following:

1. We have corrected all the inappropriate format of gene acronyms;
2. Our title says “Is MTHFD1 polymorphisms rs 2236225 (c.1958G>A)…”, which refers to a specific SNP in accordance with the content;
3. The traditional meta-analysis focuses on the clinical RCTs, which aims to reveal the relevance between specific interruption and clinical effects. A complete assessment standard for the included RCT reports has been widely accepted and used. While our study material is totally different from RCTs thus there is no clear assessment standard to refer. So we do not discuss the quality analysis. But in order to provide the readers an assessment standard we provide the tables 2 in supplementary materials for reference.
4. We agree that the usual method to evaluate publication bias is not suitable for our study because of the limited amount, which reduces the reliability of our conclusion. We have declared that in the discussion of our updated version.
5. Actually the Discussion is an overview of the article, which includes and further explains the content above. While in avoidance of repetition we modified and simplified the first and second paragraph of the Discussion.
6. Two of our included studies only analyzed the maternal genotype, one study reported the mother and children genotype and three took samples from children and their parents. The gene samples from mother were too scarce to be representative and to explain our results. We do recommend more samples from parents in the future studies, which is significant for the early stage diagnose, as the current technology can only diagnose CLP in the midterm even later in the pregnancy.

# Peer review: Referee response for version 1

**DOI:** 10.5256/f1000research.6892.r8906

**Published:** 2015-06-17

**Authors:** Jingtan Su

**Affiliations:** 1 Center for Craniofacial Molecular Biology, University of Southern California, Los Angeles, CA, USA

Non-syndromic cleft of the lip and/or palate (NSCL/P) is considered closely related to genetic and environmental factors. As a key gene in the metabolism of folic acid which is associated to high risk of NSCL/P, methylenetetrahydrofolate dehydrogenase (MTHFD1) polymorphisms rs 2237225 (c. 1958G>A) may be associated with the susceptibility of NSCL/P. However, controversial conclusions on this association have been reported by different groups. This paper conducted a systematic review and meta-analysis of eligible case-control studies which is necessary before we go forward to larger sample size studies.

This paper is scientifically sound, clear and well-organized. However, these following revisions may make it more interesting.

1. Page 6, Figure 1, Eligibility section, the number of full-text articles excluded with reasons should be 5.
2. The lack of folic acid will result in many health problems such as neural tube defects, macrocytic anemia, mental depress and so on, not only NSCL/P. MTHFD1 is important in the metabolism of folic acid. Why is it suggested that MTHFD1 polymorphisms may be associated with the susceptibility of NSCL/P, but not neural tube defects ( Meng *et al., *2015) or other diseases ( Weiner *et al.*, 2014; Silva *et al.*, 2011)?
3. It is mentioned in the Discussion section that controversial results about the MTHFD1 polymorphism rs2236225 (c. 1958G>A) have been reported in different articles. A brief description about the controversial results and their conclusions will make the discussion more interesting.
4. MTHFR, MTR, AND MTRR which are involved in the metabolism of folic acid are reported to be associated with high risk of NSCL/P. Why MTHFD1 which is also involved in the metabolism of folic acid shows no significant association with susceptibility to NSCL/P? It’s due to the sample size or the limit of technique, or it is the truth? A brief discussion on this will be interesting.

I have read this submission. I believe that I have an appropriate level of expertise to confirm that it is of an acceptable scientific standard.

# Response

**Published:** 2015-11-26

**Authors:** Feng Chen

**Affiliations:** School of Stomatology, Peking University, China

Thank you. We have studied your valuable comments and revised the manuscript according to your suggestions. The point to point responds are listed as following:

1. We have corrected the error, thank you;
2. Neural tube defects and NSCL/P is similar in the origin of development, while the different relationship with MTHFD1 may suggest the different pathogenesis. Maybe each part of organ has a specific different folic acid metabolic pathway in their development.
3. We are so glad to follow your advice. In all of the included 8 studies, 7 did not show significant difference between the case and control groups. 1 study reported the case group showed closer relationship with MTHFD1 polymorphisms rs 2236225 (c.1958G>A). A brief description of this controversy is also included in this article.
4. All 10 studies containing 6216 samples (2959 cases and 3257 controls) were analyzed in our article. Limited to the size of simple, our result cannot give a certain conclusion about the relationship of MTHFD1 and NSCL/P and won’t deny the possibility of an actual relationship. The heterogeneity exists in the research method, sample source area and maternal or children genotypes. The incident rate and clinical manifestation differ in various areas. Different research method would lead to diverse accuracy even controversial conclusion. Thus we are still not sure about the association between MTHFD1 and NSCL/P and further exploration is needed. We also discussed this matter in our article.
